# A simulated annealing heuristic for maximum correlation core/periphery partitioning of binary networks

**DOI:** 10.1371/journal.pone.0170448

**Published:** 2017-05-09

**Authors:** Michael Brusco, Hannah J. Stolze, Michaela Hoffman, Douglas Steinley

**Affiliations:** 1 Department of Marketing, Florida State University, Tallahassee, Florida, United States of America; 2 Business and Economics Department, Wheaton College, Wheaton, Illinois, United States of America; 3 Department of Psychological Sciences, University of Missouri, Columbia, Missouri, United States of America; Universitat Rovira i Virgili, SPAIN

## Abstract

A popular objective criterion for partitioning a set of actors into core and periphery subsets is the maximization of the correlation between an ideal and observed structure associated with intra-core and intra-periphery ties. The resulting optimization problem has commonly been tackled using heuristic procedures such as relocation algorithms, genetic algorithms, and simulated annealing. In this paper, we present a computationally efficient simulated annealing algorithm for maximum correlation core/periphery partitioning of binary networks. The algorithm is evaluated using simulated networks consisting of up to 2000 actors and spanning a variety of densities for the intra-core, intra-periphery, and inter-core-periphery components of the network. Core/periphery analyses of problem solving, trust, and information sharing networks for the frontline employees and managers of a consumer packaged goods manufacturer are provided to illustrate the use of the model.

## Introduction

The problem of partitioning a set of actors into two clusters, core and periphery, is a well-studied problem in the social network literature [1–8]. Excellent recent reviews of core/periphery analysis are available in [9, 10]. Conceptually, the goal is to partition the actors so as to maximize the intra-core density, while minimizing the intra-periphery density. This goal can be operationalized in different ways. One approach employs a continuous model for developing a core-to-periphery continuum whereby a degree “coreness” is established for each actor [3, 5]. However, the continuous approach does not explicitly assign actors to core and periphery subsets. Contrastingly, the discrete core/periphery approaches explicitly allocate the actors into mutually exclusive and exhaustive subsets. Throughout the remainder of this paper, attention is restricted to discrete core/periphery methods.

There are a variety of criterion functions that can be used for discrete core/periphery partitioning. Examples include Hamming distances [2, 11] and counts of inconsistencies relative to the ideal structure [4, 12, 13]. One criterion that has considerable appeal is the maximization of the correlation between an ideal and observed structure associated with intra-core and intra-periphery ties. This criterion, which is the focus of our paper, was originally proposed in [1] and is incorporated in the Ucinet software system [11]. The heuristic procedure designed in [1] for obtaining solutions to the maximum correlation core/periphery partitioning problem is a variation of the genetic algorithm [14].

The study [2] compared four approaches for the maximum correlation core/periphery partitioning problem: (i) the Ucinet genetic algorithm [1, 9], (ii) a genetic algorithm implementation available as a built-in function in *Mathematica*, (iii) a simulated annealing algorithm [15] available as a built-in function in *Mathematica*, and (iv) a *Mathematica* coding of a relocation/exchange algorithm [16]. The principal conclusions in [2] were that the *Mathematica* implementations of the genetic algorithm and Kernigan-Lin heuristic were more robust than the Ucinet genetic algorithm and simulated annealing; however, these comparisons were limited to small networks with *n* = 20 or fewer actors.

It is important to establish from the outset that our goal is not to tout maximum correlation core/periphery partitioning as the best approach for analyzing core/periphery structure. Indeed, there are many other approaches available in the literature that are better-suited for certain applications. Nevertheless, maximum correlation core/periphery partitioning does have a history in the literature and is available in a popular software program. Accordingly, the purpose of our paper is to show that, contrary to earlier findings [2], an efficient implementation of simulated annealing can be a viable approach to this problem. The process for accomplishing this purpose is twofold. First we present a formal statement of the maximum correlation core/periphery partitioning problem originally proposed in [1] and also studied in [2]. An important aspect of this presentation is the efficient computation of the correlation between two binary vectors [17] and the process for capitalizing on this efficiency in a heuristic implementation. Although the precise computational details of prior implementations is not entirely clear, there is some evidence that previous approaches to calculating the correlation measure have been somewhat inefficient. For example, in the study reported in ([2], p. 176), it was noted that trial solutions were evaluated by “…recomputing the fitness from scratch and subtracting it from the old fitness.” The method for computing correlation between binary vectors that is described herein is appreciably more efficient, and this is a profound advantage when tackling large instances of the maximum correlation core/periphery partitioning problem.

Second, a simulated annealing heuristic using a reciprocal cooling scheme is proposed, which requires the selection of only one parameter: the number of iterations. Although the results in [2] indicated that the *Mathematica* implementation of simulated annealing performed poorly, we believe that our simulated annealing approach using reciprocal cooling is much more effective. To demonstrate this effectiveness, we ensure that, for modestly-sized empirical networks from the literature, the proposed algorithm consistently produces the same criterion function value across multiple restarts. Moreover, the simulated annealing algorithm is evaluated across a broad range of synthetic networks that are much larger than those considered in previous studies [2–5]. Specifically, the synthetic networks range in size from 500 to 2000 actors and have different levels of intra-core, intra-periphery, and inter-core-periphery densities. The proposed algorithm is used to conduct core/periphery analyses for problem solving, trust, and information sharing networks corresponding to frontline employees of a consumer packaged goods manufacturer. Finally, the simulated annealing algorithm is applied to a larger real-world network and its performance is compared to results obtained using Ucinet [11].

The next section presents the formal statement of the maximum correlation core/periphery partitioning problem that is studied in this paper. This is followed by a description of the proposed simulated annealing heuristic. The new heuristic is then applied to several small empirical networks. Subsequently, a simulation study is offered to evaluate the computational efficiency of the simulated annealing heuristic under various data conditions. The heuristic is then applied to three networks associated with employees from a consumer packaged goods manufacturer. The paper concludes with a brief summary, along with limitations and extensions for future research.

## Maximum correlation core/periphery partitioning

### Model formulation

The simulated annealing algorithm for the maximum correlation core/periphery partitioning of binary networks has sufficient flexibility to handle either symmetric or asymmetric network matrices. The formulation of the underlying optimization problem assumes that *n* is the number of actors in the network and that *T* = {1, 2, …, *n*} is the set of indices for those actors. An *n* × *n* binary matrix **A** = [*a*_*ij*_], of network ties among the actors is assumed to be available, where *a*_*ij*_ = 1 if there is a tie from actor *i* to actor *j* and *a*_*ij*_ = 0 otherwise, for 1 ≤ *i* ≠ *j* ≤ *n*. As is common in most network applications [18], the main diagonal of **A** is ignored in the analyses. The term ∏ is used to denote the set of all partitions of the *n* actors into core and periphery subsets, and π = {*T*_1_, *T*_0_} is a partition from ∏, where *T*_1_ is the set of actors assigned to the core and *T*_0_ is the set of actors assigned to the periphery.

The number of actors in core and periphery of partition π are denoted as *n*_*C*_ = |*T*_1_| and *n*_*P*_ = |*T*_0_|, respectively. Likewise, the number of intra-core and intra-periphery dyads for partition π are defined as *d*_*C*_(π) = $n_{C}^{2}-n_{C}$ and *d*_*P*_(π) = $n_{P}^{2}-n_{P}$, respectively. The total number of intra-core and intra-periphery dyads for partition π is *d*(π) = *d*_*C*_(π)+ *d*_*P*_(π). The total number of intra-core violations for partition π (zeros in core submatrix) is:

$$v_{C}(\pi )=\sum_{\{i\neq j\}\in T_{1}}\left(1-a_{ij}\right).$$ (1)

The total number of intra-periphery violations (ones in periphery submatrix) for partition π is:

$$v_{P}(\pi )=\sum_{\{i\neq j\}\in T_{0}}a_{ij}.$$ (2)

We also defined **x**(π) as a vector corresponding to an ideal core/periphery structure for partition π, where the first $n_{C}^{2}-n_{C}$ elements of the vector are ones (i.e., a perfectly connected core) and the last $n_{P}^{2}-n_{P}$ elements of the vector are zeros (i.e., a perfectly unconnected periphery). Similarly, the vector **y**(π) corresponds to the observed core/periphery structure for partition π, where the first $n_{C}^{2}-n_{C}$ elements are the values of *a*_*ij*_ among all pairs of actors {*i* ≠ *j*} ϵ *T*_1_ and the last $n_{P}^{2}-n_{P}$ elements are the values of *a*_*ij*_ among all pairs of actors {*i* ≠ *j*} ϵ *T*_0_. The term *r*_*XY*_(π) represents the bivariate correlation between the vectors **x**(∏) and **y**(π). The optimization problem for the maximum correlation core/periphery partitioning of binary networks as proposed in [1] is:

$$\text{Maximize}\;:\;r_{XY}(\pi )$$ (3)

Subjectto:π∈Π. (4)

Succinctly, Eq (4) ensures that a partition (π) is selected from the set of all partitions (∏) with the goal of maximizing the correlation criterion function (3).

### Efficient computation of *r*_*XY*_(π) and fast updating

Given the substantial length of the **x** and **y** vectors, it would be computationally demanding to compute *r*_*XY*_(π) from scratch (e.g., using common formulas for the Pearson correlation coefficient) each time a new partition was constructed in a heuristic algorithm. Fortunately, the correlation between the two binary vectors **x** and **y** can be computed efficiently using the constants defined above. This is important because it allows for rapid recomputation of *r*_*XY*_(π) using simple updates to these constants each time the partition is modified by moving an actor from the core to the periphery or vice versa. The formulas for computing the correlation are as follows:

The variance of **x** for partition π is computed as:

$$s_{X}^{2}(\pi )=\left(\frac{d_{C}(\pi )}{d(\pi )}\right)\left(\frac{d_{P}(\pi )}{d(\pi )}\right).$$ (5)

The variance of **y** for partition π is computed as:

$$s_{Y}^{2}(\pi )=\left(\frac{\left(d_{C}(\pi )-v_{C}(\pi )+v_{P}(\pi )\right)}{d(\pi )}\right)\left(\frac{\left(d_{P}(\pi )-v_{P}(\pi )+v_{C}(\pi \right)}{d(\pi )}\right).$$ (6)

The covariance between **x** and **y** for partition π is computed as:

$$s_{XY}^{2}(\pi )=\left(\frac{\left(d_{C}(\pi )-v_{C}(\pi )\right)}{d(\pi )}\right)-\left(\frac{d_{C}(\pi )}{d(\pi )}\right)\left(\frac{\left(d_{C}(\pi )-v_{C}(\pi )+v_{P}(\pi \right)}{d(\pi )}\right).$$ (7)

The correlation between **x** and **y** for partition π is computed as:

$$r_{XY}(\pi )=\left(\frac{s_{XY}^{2}}{\sqrt{s_{X}^{2}s_{Y}^{2}}}\right).$$ (8)

As an illustration of the formulae for computing the correlation measure, we consider an asymmetric core/periphery solution originally reported in [1], and later confirmed in [2]. The binary asymmetric network matrix corresponds to co-citation ties among *n* = 20 social work journals [19]. The core/periphery partition, π, reported in ([1], p. 385) consists of *n*_*C*_(π) = 6 core journals and *n*_*P*_(π) = 14 periphery journals. Applying the formulas in the previous subsection yields *d*_*C*_(π) = 6^2^−6 = 30, *d*_*P*_(π) = 14^2^−14 = 182, and *d*(π) = 30 + 182 = 212. From the table published in ([1], p. 385), it is straightforward to count *v*_*C*_(π) = 2 intra-core violations (zeros in the intra-core submatrix) and *v*_*P*_(π) = 8 intra-periphery violations (ones in the intra-periphery submatrix–ignoring the main diagonal). From Eq (5), the variance for the ideal structure is $s_{X}^{2}(\pi )=\left(\frac{30}{212}\right)\left(\frac{182}{212}\right)=.121485$. Likewise, from Eq (6), the variance for the observed structure is $s_{Y}^{2}(\pi )=\left(\frac{\left(30-2+8\right)}{212}\right)\left(\frac{(182-8+2}{212}\right)=.140975$. The covariance is computed as $s_{XY}^{2}(\pi )=\left(\frac{\left(30-2\right)}{212}\right)-\left(\frac{30}{212}\right)\left(\frac{\left(30-2+8\right)}{212}\right)=.108046$ using Eq (7). Finally, the correlation is computed using Eq (8) as $r_{XY}(\pi )=\left(\frac{.108046}{\sqrt{\left(.121485)(.140975\right)}}\right)=.82561$. When rounded to three decimal places, this correlation value comports to the one reported in ([1], p. 385) and is identical to the five-decimal place value reported in ([2], p. 171).

The proposed computational approach is particularly valuable for rapidly assessing the impact of neighborhood search operations applied to a given partition. To describe the process for updating the correlation measure, assume that a new partition π′ is constructed by moving actor *h* from the core (*T*_1_) to the periphery (*T*_0_). This move results in *n*_*C*_(π′) = *n*_*C*_(π)– 1 and *n*_*P*_(π′) = *n*_*P*_(π) + 1. The formulas in the previous subsection can then be directly applied to obtain *d*_*C*_(π′) and *d*_*P*_(π′), respectively. An update of the intra-core violations is provided as follows:

$$v_{C}(\pi^{\prime })=v_{C}(\pi )-\sum_{\{j\neq h\}\in T_{1}}\left(1-a_{hj}\right)+(1-a_{jh})).$$ (9)

Essentially, Eq (9) removes the contribution to intra-core violations that stem from actor *h*’s relationship to all other actors in the core, which is appropriate given *h*’s removal from the core. Likewise, because *h* is moved into the periphery, it is necessary to increase the intra-periphery violations (ones in the periphery submatrix) that might arise from *h*’s inclusion in the periphery. This is accomplished as follows:

$$v_{P}(\pi^{\prime })=v_{P}(\pi )+\sum_{\{j\neq h\}\in T_{0}}(a_{hj}+a_{jh}).$$ (10)

After making these rapid updates, the correlation for the trial partition π′ is readily computed. In the case where the new partition π′ is realized by moving actor *h* from the periphery (*T*_0_) to the core (*T*_1_), the result is *n*_*C*_(π′) = *n*_*C*_(π) + 1 and *n*_*P*_(π′) = *n*_*P*_(π)– 1. The values of *d*_*C*_(π′) and *d*_*P*_(π′) would be updated accordingly. An update of the intra-core violations is provided as follows:

$$v_{C}(\pi^{\prime })=v_{C}(\pi )+\sum_{\{j\neq h\}\in T_{1}}\left((1-a_{hj}\right)+(1-a_{jh})).$$ (11)

Eq (11) augments the contribution to intra-core violations that stem from actor *h*’s inclusion in the core via examination of its ties to all other actors in the core. In a similar manner, because *h* is removed from the periphery, it is necessary to decrease the intra-periphery violations (ones in the periphery submatrix) in accordance with *h*’s ties to other actors in the periphery. This is accomplished as follows:

$$v_{P}(\pi^{\prime })=v_{P}(\pi )-\sum_{\{j\neq h\}\in T_{0}}(a_{hj}+a_{jh}).$$ (12)

## Methods

### Existing procedures

Solution procedures for the maximum correlation core/periphery partitioning problem can be divided into two categories: (i) exact and (ii) heuristic. Exact methods are assured to produce a globally optimal solution to the problem posed by Eqs 6 and 7, whereas heuristic methods do not provide assurance. One exact solution approach is to explicitly generate all partitions in ∏ and select the one that provides the largest value for *r*_*XY*_(π). The feasibility of this approach is likely restricted to networks with 30 or fewer actors, as the number of partitions in ∏ exceeds one billion when *n* = 30. An alternative exact approach is to employ branch-and-bound methods [4]. Unfortunately, the efficient design of strong bounds for the maximum correlation criterion function is not trivial and, even if good bounds could be established, the size of networks that could be tackled would remain modest.

Heuristic procedures for the maximum correlation core/periphery problem include the genetic algorithm available in Ucinet [1, 11], as well the exchange algorithm, simulated annealing, and genetic algorithm approaches considered in [2]. Other metaheuristics such as tabu search [20], variable neighborhood search [21] would also be viable. In light of the fact that the built-in simulated annealing program in *Mathematica* performed so poorly in the study completed in [2], we sought to design an effective simulated annealing implementation for the maximum correlation core/periphery partitioning problem. This method is described in the next subsection

### Proposed simulated annealing heuristic

Simulated annealing was independently developed in [22] and [23] as a heuristic procedure for combinatorial optimization problems. The method is based on an analogy to the metallurgical process of annealing in statistical mechanics [24], whereby a better final energy state is often achieved by periodically re-heating the metal during the cooling process. The adaptation of this concept for optimization problems is that, during a local-search process, periodically accepting a trial solution (π′) that has a criterion function value that is worse than that of the incumbent solution (π) will ultimately lead to the achievement of a better final criterion function value. The probability of accepting a solution that worsens the criterion function is controlled by the extent of the worsening, which in our context is *r*_*XY*_(π′)–*r*_*XY*_(π) < 0, as well as the current temperature (τ) of the system. During the execution of the algorithm, the value of τ is systematically reduced to lower the probability that inferior solutions will be accepted. The process for reducing τ is known as the cooling scheme.

There are a variety of possible cooling schemes that can be used for simulated annealing, including the linear, exponential (or geometric), reciprocal, and logarithmic approaches (see [25–27]). In this paper, we use the reciprocal cooling scheme. Given an initial temperature, τ(1), and an upper limit on the number of trial solutions generated, ξ, the temperature, τ(*q*), for trial solution *q* is computed as τ(*q*) = τ(1)/*q* using the reciprocal cooling scheme.

A natural choice for the initial temperature is τ(1) = 1 because the maximum possible value of the correlation measure is one. The relocation of an actor from the core to the periphery (or vice versa) will typically produce a very small change (much less than one) in the criterion function, particularly for larger networks. Therefore, in the early stages of the algorithm with a larger cooling parameter, almost every trial solution will be accepted regardless of whether it improves of worsens the criterion function value. However, by trial solution *q* = 10,000, the value of τ(10,000) = 0.0001, and the probability of accepting a solution that worsens the criterion function only slightly is quite small.

If the initial temperature is fixed at τ(1) = 1, then the only user-specified parameter for the algorithm is ξ. If ξ is chosen too small, then the algorithm might not reach temperatures that are low enough to ensure convergence to a good solution. On the other hand, choosing a value of ξ that is too large can be computationally wasteful, as no inferior trial solutions will be accepted once the temperature is too small. We have found that a range of 100,000 ≤ ξ ≤ 1,000,000 trial solutions seems to generally perform well for a diverse range of network sizes.

**Fig 1:**
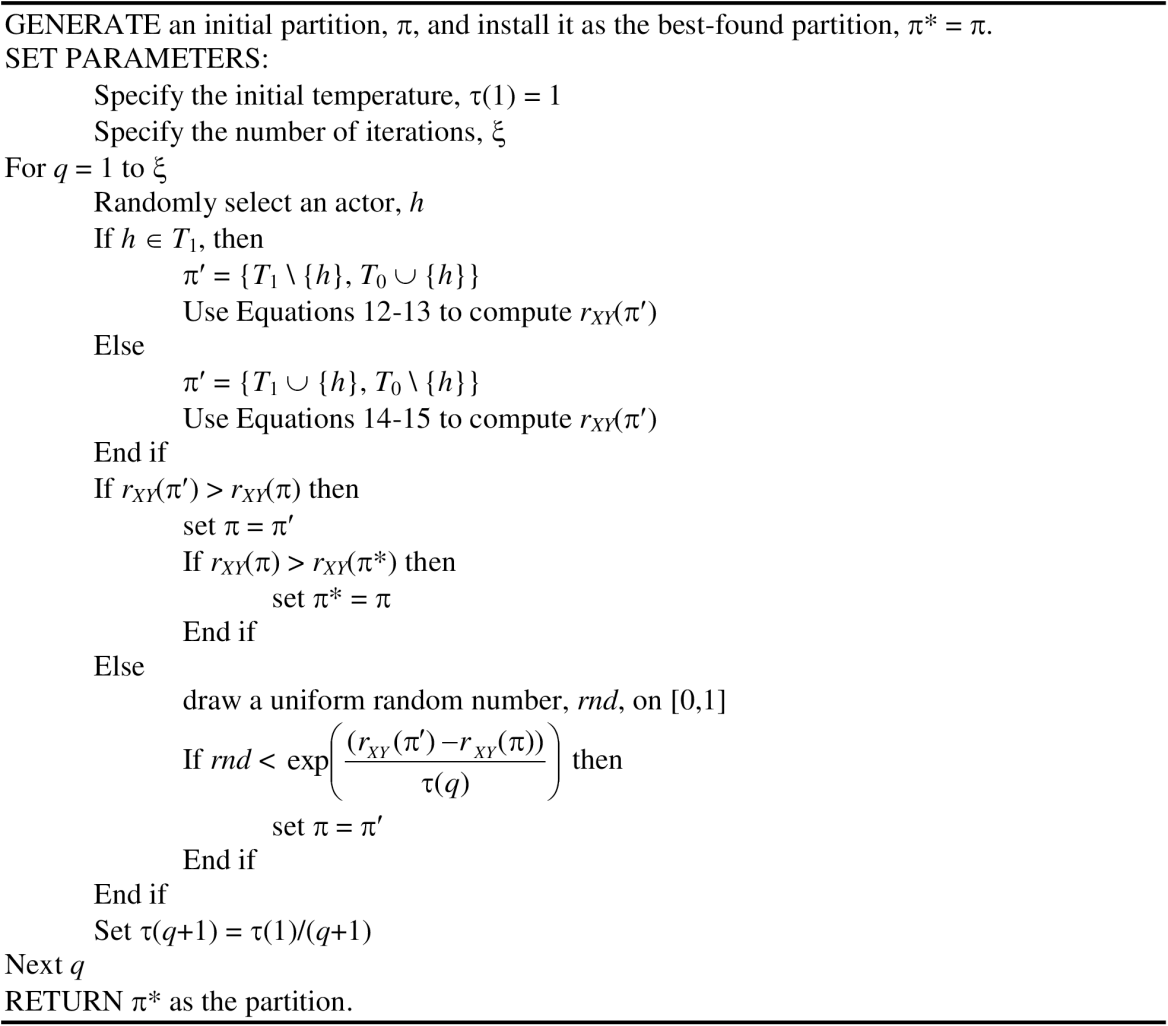
Simulated annealing (reciprocal cooling) pseudo code.

Fig 1 provides a pseudo code for our implementation of simulated annealing with a reciprocal cooling scheme. An initial partition, π, is randomly generated by assigning each actor to either the core or the periphery based on a uniform distribution with 50% probability for each subset. This initial partition is then installed as the best-found partition, π*. The algorithm then generates the selected number of trial solutions (ξ). For each trial solution, an actor is randomly selected, and the cluster membership of that actor is changed from core to periphery or periphery to core as appropriate. The computation of *r*_*XY*_(π′) for trial solution π′ is performed using the Eqs 8–13. If *r*_*XY*_(π′) ≥ *r*_*XY*_(π), then π′ replaces π as the incumbent solution and a check is also made to see if this new incumbent has a greater correlation index and should therefore replace the current best-found solution π*. If *r*_*XY*_(π′) < *r*_*XY*_(π), then π′ replaces π as the incumbent solution with a probability of $\exp\left(\frac{\left(r_{XY}(\pi^{\prime })-r_{XY}\left(\pi \right)\right)}{\tau (q)}\right)$.

## Application to empirical matrices

### Selected networks

Our principal focus in this section is to ensure that the simulated annealing algorithm will consistently provide good solutions for modestly-sized networks. Four networks were selected from the literature for analysis. The first two networks correspond to co-citation network data for 20 social work journals during the period from 1985 to 1986 [19]. These network data have been explored in several previous studies [1–5, 11]. The first network matrix corresponds to a symmetric dichotomization ([1], p. 382) of the social work journal co-citation data, whereas the second is the asymmetric dichotomized data ([1], p. 385).

The third network matrix corresponds to the Kansas Search and Air Rescue Communication (Kansas SAR) study that was originally reported in [28]. The asymmetric dichotomized matrix was adapted from the one published in ([13], p. 55) and reflects message sending and receiving ties among the *n* = 20 network agencies. The fourth matrix is a 28 × 28 asymmetric dichotomized co-citation matrix among statistical and quantitative psychology (and other social science) journals. This matrix was taken from a study in [4], which adapted the network from co-citation data published in ([29], p. 547).

### Analysis and results

The simulated annealing algorithm was programmed in Fortran 90 and implemented on a on a desktop computer using an Intel ® Core ™ i7-4790 CPU @ 3.6GHz with 8 GB of RAM. The algorithm was applied to each of the four empirical network matrices using ξ = 100,000 as the number of trial solutions. For each network matrix, implementation of the algorithm was repeated 10 times using a different random initial partition for each repetition. The primary performance measure was the *attraction rate*, which represents the number of repetitions for which the best criterion function value was obtained. The secondary performance measure was the maximum computation time across all 10 restarts. The results are reported in Table 1.

**Table 1:** Attraction rates and computation times for empirical network matrices.

Network	Size	*r*_*XY*_(π)	Attraction rate	Maximum computation time
Social work journal co-citations				
Symmetric, dichotomized	*n* = 20	.85959	10 out of 10	.01
Sources: [1, 17]				
Social work journal co-citations				
Asymmetric, dichotomized	*n* = 20	.82561	10 out of 10	.01
Sources: [1, 17]				
Statistics/Psychology co-citations				
Asymmetric, dichotomized	*n* = 28	.50694	10 out of 10	.01
Sources: [4, 27]				
Kansas SAR Communications				
Asymmetric, dichotomized	*n* = 20	.71047	10 out of 10	.01
Sources: [11, 26]				

Note–‘Attraction Rate’ is the number of repetitions (out of 10) for which the reported criterion value was achieved. The maximum computation times are in seconds.

The results in Table 1 indicate that, for each of the four network matrices, the simulated annealing algorithm produced the same criterion function value for all 10 repetitions. This consistency is important because it shows that, for modestly-sized networks, the algorithm is not highly sensitive to the initial starting partitions. The maximum computation times reported in Table 1 are also modest. Across all matrices and repetitions, the maximum computation time across the 10 restarts was only .01 seconds.

For the two social work co-citation networks, the partitions obtained correspond to those originally published in ([1], pp. 382, 385) and later confirmed in [2]. For the psychological/statistical network, the core was very similar to the one obtained and displayed in ([4], p. 15, Table 2) using a criterion function that summed the number of (non-diagonal) zeros in the core and the number of (non-diagonal) ones in the periphery. The core from the partition reported in [4] consisted of nine journals: {*Psychometrika*, *British Journal of Mathematical and Statistical Psychology*, *Multivariate Behavioral Research*, *Psychological Bulletin*, *Annals of Statistics*, *Biometrics*, *Biometrika*, *Journal of the American Statistical Association*, *Journal of the Royal Statistical Society B*}. The first four members of the core are statistical psychology journals and last five are elite statistical outlets. The partition we obtained using the maximum correlation criterion function added the journal *Applied Statistics* to the core. The practical merit of the inclusion of this journal in the core is debatable, as it is more applied in nature relative to the other five statistical journals.

**Table 2:** Summary of simulation results.

		MRCD Results		MPCD Results		MPBF Results	
Feature	Level	100,000	500,000	100,000	500,000	100,000	500,000
Number of actors	*n* = 500	.00003	.00000	.02947	.00053	25.00	94.44
	*n* = 1000	.00028	.00001	.25364	.00432	13.89	48.61
	*n* = 2000	.00147	.00003	1.47529	.03155	11.11	20.83
Percentage of	5%	.00101	.00002	1.24427	.02427	1.39	40.28
actors in core	10%	.00067	.00002	.47884	.01090	1.39	44.44
	20%	.00009	.00000	.03529	.00123	47.22	79.17
Intra-core density	70%	.00062	.00001	.60961	.01304	12.04	51.85
	80%	.00057	.00001	.56266	.01122	21.30	57.41
Intra-periphery	20%	.00057	.00001	.45406	.00947	20.37	57.41
density	30%	.00061	.00001	.71821	.01480	12.96	51.85
Inter core-periphery	40%	.00054	.00001	.70895	.01239	25.93	62.96
density	60%	.00065	.00002	.46332	.01188	7.41	46.30
Overall		.00059	.00001	.58613	.01213	16.67	54.63

Note–the results in the table correspond to 100,000 and 500,000 temperature reductions relative to 1 million:

MRCD–mean raw correlation differences

MPCD–mean percentage correlation differences

MPBF–mean percentage of best-found correlation values

**Fig 2:**
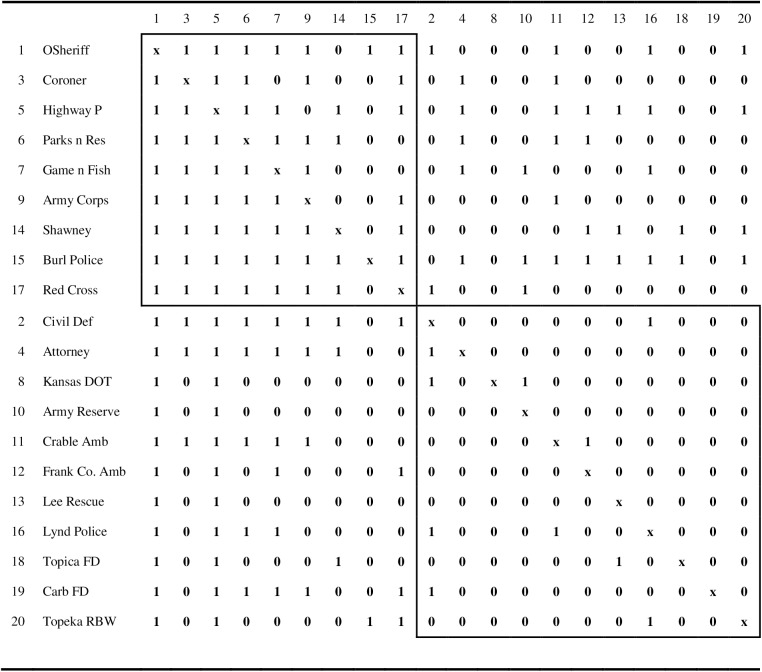
The core/periphery partition for the Kansas SAR network obtained using the simulated annealing algorithm. The naming system for the agencies is from [11, p. 54), and the rows are the ‘sending’ agencies, whereas the columns are the ‘receiving’ agencies. The two boxes contain the intra-core and intra-periphery elements.

The core/periphery partition for the Kansas SAR network is displayed in Fig 2. The value of the criterion function for this partition is *r*_*XY*_(π) = .71047. The naming convention for the agencies in the network was provided in ([11], p. 54). The core consists of nine agencies, most of which exhibit a high degree of symmetry with respect to their sending and receiving of messages. The notable exceptions are ‘Shawney’ and ‘Burl Police’, which send messages to most of the other core members, but do not receive messages from the other core members.

### A Simulation study

#### Simulation design

Simulated annealing proved effective for the modestly-sized networks in the previous section. However, our goal here is to provide a more robust simulation-based evaluation of the method across a set of much larger networks with various core sizes and density characteristics. Five data features were manipulated in the experimental design. The first feature, the number of actors, was tested at three levels: *n* = 500, 1000, and 2000. The second feature controlled the relative sizes of the core and periphery subsets, and was tested at three levels: (a) the core size is .05*n* (i.e., 5% of the actors in the core), (b) the core size is .10*n*, and (c) the core size is .20*n*. We selected these levels because the core is typically smaller than the periphery. The third, fourth, and fifth design features corresponded to the intra-core density, intra-periphery density, and inter-core-periphery density, respectively. The levels of the third design feature were 80% and 70%. The levels of the fourth design were 30% and 20%. The levels of the fifth design feature were 40% and 60%.

A fully-crossed design was employed, resulting in 3 × 3 × 2 × 2 × 2 = 72 cells. Three problem replications were generated in each cell, which yielded a total of 216 test problems in the experiment. The simulated annealing heuristic was applied to each of the 216 test problems for three different numbers of trial solutions: (i) ξ = 100,000, (ii) ξ = 500,000, and (iii) ξ = 1,000,000. The criterion function values and computation times were collected for each of the three runs of the simulated annealing heuristic for each test problem.

The results for ξ = 1,000,000 trial solutions provides a benchmark for evaluating the results at the other two levels of ξ. Because the results for the simulated algorithm cannot worsen as ξ is increased, the best-found correlation criterion function value for each test problem is realized for ξ = 1,000,000. For the settings of ξ = 100,000 and ξ = 500,000, two performance measures are computed for each test problem: (i) the raw deviation between the measured correlation value and best-found correlation value for ξ = 1,000,000, and (ii) the raw deviation measured expressed as a percentage of the best-found correlation value for ξ = 1,000,000. From these performance measures, three summary measures are computed, both overall and for different levels of the design features: (i) mean raw correlation deviation (MRCD), (ii) mean percentage correlation deviation (MPCD), and (iii) mean percentage of best-found correlation values (MPBF).

### Simulation results

The computation times for the simulated annealing algorithm were affected by two conditions: the number of actors (*n*) and the number of trial solutions (ξ). Computation times ranged from roughly one-half second for the test problems with *n* = 500 and ξ = 100,000, to roughly 30 seconds for test problems with *n* = 2000 and ξ = 1,000,000. These times are modest and suggest that the algorithm is computationally feasible for a sufficiently large number of trial solutions (e.g. ξ ≥ 1,000,000) for networks with several thousand vertices. Nevertheless, it should be noted that computation time is not a linear function of *n*. For each value of ξ, the average computation time for the *n* = 1000 test problems is slightly more than *double* the average of the *n* = 500 test problems. However, the average computation time for the *n* = 2000 test problems is slightly more than *triple* the average of the *n* = 1000 test problems.

Table 2 reports the MRCD, MPCD, and MPBF values for each level of each design feature of the simulation experiment. The overall (across all test problems) averages are also reported at the bottom of the table. The overall MRCD values for ξ = 100,000 and ξ = 500,000 were .00059 and .00001, respectively. Moreover, across all test problems, the maximum difference between the correlation criterion function measured for ξ = 100,000 and ξ = 1,000,000 was only .00289 (.04981 vs. .05270). Likewise, across all problems, the maximum difference between the correlation criterion function measured for ξ = 500,000 and ξ = 1,000,000 was only .00011 (.13111 vs. .13122). The overall MPCD values for ξ = 100,000 and ξ = 500,000 were .58613% and .01213%, respectively. Across, all test problems, the maximum percentage deviations were 5.48387% and .11021% for ξ = 100,000 and ξ = 500,000, respectively. Finally, the overall MPBF values for ξ = 100,000 and ξ = 500,000 were 16.67% and 54.63%, respectively.

The intra-core density, intra-periphery density, and inter-core-periphery density design features exhibited a relatively modest effect on the MRCD, MPCD, and MPBF measures. However, these measures were more strongly affected by the levels for the number of actors and the percentage of actors in the core. Consider, for example, at *n* = 500, the implementations using ξ = 100,000 and ξ = 500,000 matched the best-found correlation values for 25.00% and 94.44% of the test problems, respectively. However, for *n* = 2000, the ξ = 100,000 and ξ = 500,000 implementations matched the best-found correlation values for ξ = 1,000,000 for only 11.11% and 20.83% of the test problems, respectively.

The ξ = 100,000 and ξ = 500,000 implementations performed reasonably well when the percentage of actors in the core was 20%, matching the best-found correlation values for 47.22% and 79.17% of the test problems, respectively. However, their attraction to the best-found solution was markedly less impressive for the smaller core size levels of 5% and 10%. For these smaller percentages of actors in the core, the ξ = 100,000 implementation only matched the best-found correlation value about 1% of the time, whereas the ξ = 500,000 implementation matched the best-found correlation roughly 40% of the time.

## Core/Periphery analyses of organizational networks

### Network relations

Three networks were used to demonstrate the simulated annealing heuristic for the maximum correlation core/periphery partitioning problem. The networks were obtained using the full roster method [18] for *n* = 25 mangers and frontline employees of a consumer packaged goods manufacturer. The 25-member bounded social group consisted of frontline truck drivers and managers assigned to a specific regional cross-dock warehouse location. One-hundred percent of the bounded social group responded to the survey, ranking each of the other members of their social network for the most rigorous recall-based elicitation of social network relations and ultimately, structure [30]. The research team administered the surveys directly to the frontline employees and management team at a scheduled monthly meeting.

The three relations examined were: (i) *problem solving*–that is, identifying those from whom assistance is sought when a problem arises, (ii) *trust*–identification of those persons from whom advice is trusted, and (iii) *information sharing*–identification of those persons from whom information about upcoming store promotions was sought. The three resulting network matrices were asymmetric and, for each network, the rows of the corresponding network correspond to the tie-senders (the respondents) and the columns correspond to the tie-receivers (the alters).

### Analysis and results

**Fig 3:**
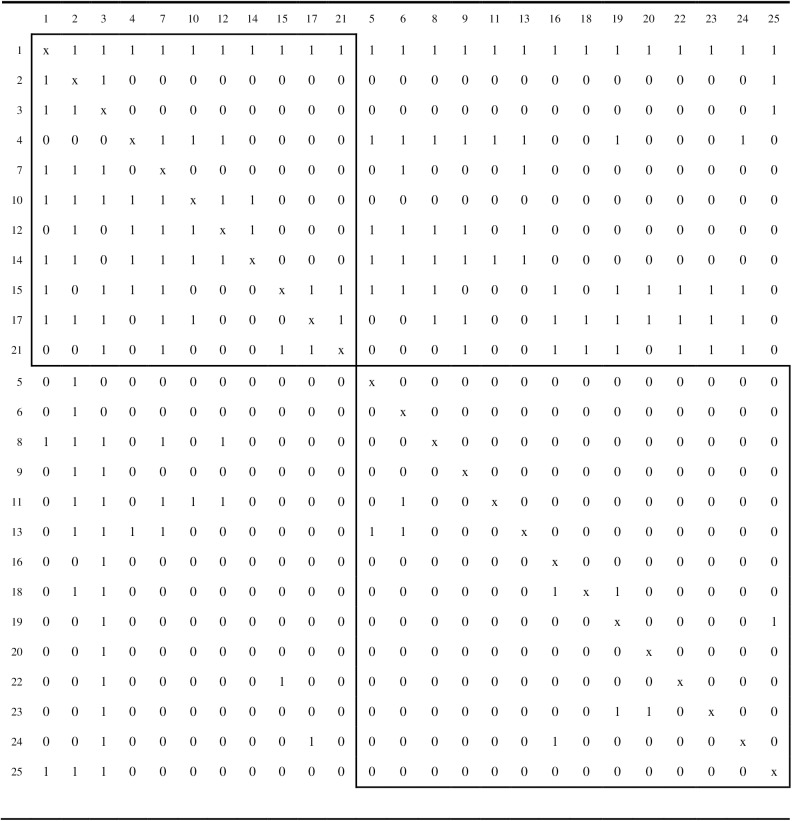
The core/periphery partition for the problem solving network. The two boxes contain the intra-core and intra-periphery elements.

**Fig 4:**
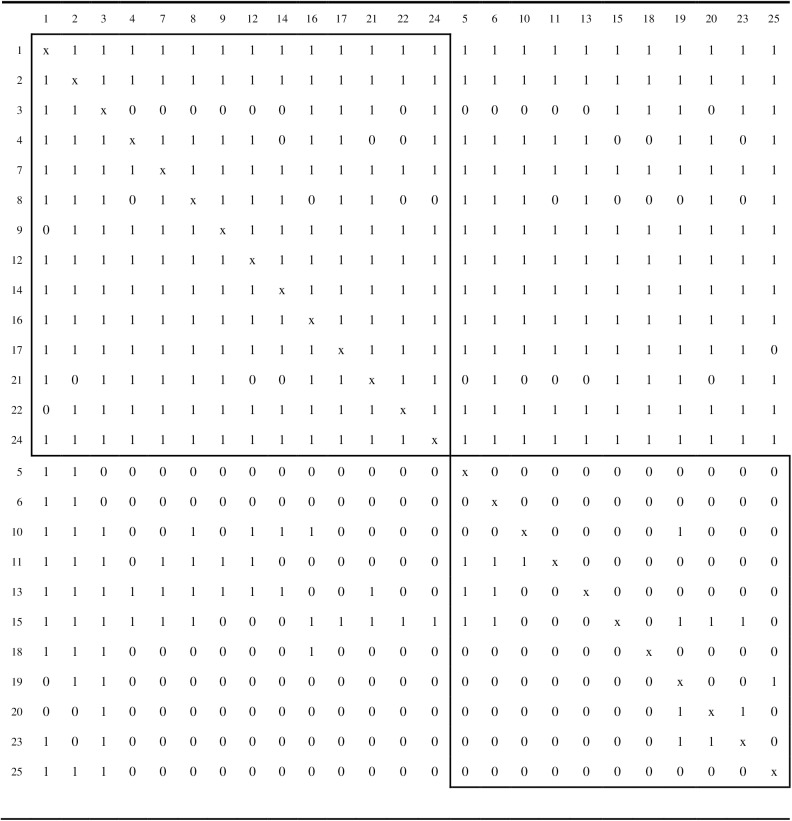
The core/periphery partition for the trust network. The two boxes contain the intra-core and intra-periphery elements.

**Fig 5:**
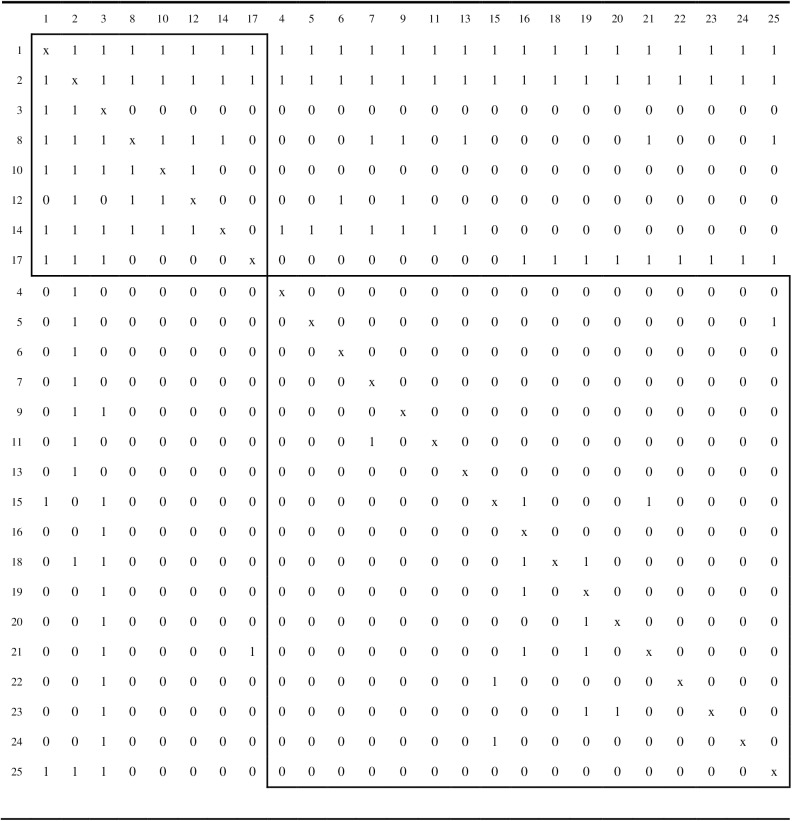
The core/periphery partition for the information sharing network. The two boxes contain the intra-core and intra-periphery elements.

Ten repetitions of the simulated annealing algorithm were applied to each of the three network matrices. For each matrix, the simulated annealing algorithm obtained the same partition for each of the 10 repetitions, which is consistent with the findings for the empirical network matrices investigated above. Although these strong attraction rates are not unequivocal proof that the global maximum for the correlation criterion function has been achieved, they do tend to support the likelihood of this result. Figs 3, 4 and 5 display the core/periphery partitions for problem solving, trust, and information sharing, respectively.

The trust network (see Fig 4) is far more dense (density of .60667) than the other two networks and, therefore, not surprisingly, has the largest of the cores (14 actors). The core/periphery partition for the trust network also has a larger correlation criterion function value, *r*_*XY*_(π) = .74630, than the other two networks. The information sharing network (see Fig 5) is the least dense (density of .22167) of the three networks and has the smallest core (8 actors). However, the core/periphery partition for the information sharing network also has a solid correlation criterion function value of *r*_*XY*_(π) = .65933. The density of the problem solving network (density of .26000) is slightly greater than that of the information sharing network, and its core is also larger at 11 actors. However, the problem solving network is the least well-structured, as it core/periphery partition has a correlation criterion value of only *r*_*XY*_(π) = .52004.

Six of the actors, {1, 2, 3, 12, 14, 17}, are present in the cores of the partitions for the problem solving, trust, and information sharing networks. Accordingly, these six actors occupy central positions for all three relational roles within the organization. Three of the actors, {4, 7, 21}, are in the cores for the partitions for the problem solving and trust networks, but not the information sharing networks. Actor 10 is in the core for the problem solving and information sharing networks, but not the trust network. Actor 8 is in the core for the trust and information sharing networks, but not the problem solving network. Four actors, {9, 16, 22, 24} appear in the core for the trust network only, and actor 15 is in the core for the problem solving network only. Nine actors,{5, 6, 11, 13, 18, 19, 20, 23, 25}, were in the periphery for all three networks.

## Efficiency and effectiveness for a larger network

To complete our analyses, we studied the efficiency and effectiveness of the simulated annealing algorithm by applying it to a larger binary network (*n* = 2114). The data correspond to a protein interaction network of yeast [31] and are available from several sources including: http://www3.nd.edu. We applied the 10 restarts of the simulated annealing algorithm to these data using ξ = 100,000. For comparison purposes, we also conducted maximum-correlation core/periphery partitioning using Ucinet [11].

Maximum-correlation core/periphery partitioning in Ucinet was accomplished using the built-in genetic algorithm with the CORR option. The algorithm required 10 hours and 47 minutes on a an Intel ® Core ™ i7-4720HQ CPU @ 2.6GHz laptop computer with 8 GB of RAM to produce a solution with a correlation of .07486. By contrast, each of the 10 restarts of the simulated annealing algorithm required less than 10 seconds to produce a solution, and the correlation values ranged from .07649 to .07712. In summary, each of the 10 restarts produced a better solution than the one found by Ucinet and did so in dramatically less time.

## Conclusions

We have presented a new simulated annealing algorithm for maximum correlation core/periphery partitioning of binary networks. The algorithm is exceptionally fast and requires minimal parameterization. A key component of the algorithm is that it uses an efficient process for updating the Pearson correlation measure between two binary vectors as trial solutions are generated. This is in contrast to some earlier implementations whereby trial solutions were evaluated by recomputing the correlation measure from scratch [2].

The algorithm was initially applied to four small empirical networks to measure its consistency across multiple repetitions. For each of the four networks, the algorithm identified the same partition for each of its 10 repetitions. These findings suggest that the relatively poor performance of simulated annealing in the comparative analyses in [2] was an artifact of the particular generic implementation in *Mathematica*, not an indication of an inherent flaw in the simulated annealing approach itself.

Next, a simulation study was completed, wherein the algorithm was applied to much larger synthetic networks ranging in size from 500 to 2000 actors. The levels of core size, intra-core density, intra-periphery density, and inter-core-periphery density were controlled in the simulation study, and criterion function performance was evaluated at three different levels for the maximum number of iterations: 100,000, 500,000, and 1 million. The results of the simulation study indicated that 500,000 was a reasonable iteration limit even for the largest networks.

The use of the algorithm was demonstrated via an application to three networks (problem solving, trust, and information sharing) corresponding to 25 frontline employees and managers for a consumer packaged goods manufacturer. As was the case for the four empirical network matrices from the literature, the new simulated annealing algorithm consistently provided the same maximum correlation criterion function value across 10 repetitions.

The algorithm was also applied to a much larger (*n* = 2114) protein interaction network from the literature. Ten restarts of the algorithm produced correlation values within a narrow range (.07649 to .07712). The computation time for each restart was less than 10 seconds. For comparative purposes, we tackled the same network using the maximum-correlation core/periphery partitioning capabilities in Ucinet. The genetic algorithm used by Ucinet required more than 10 hours to obtain a solution, and the correlation of .07486 for that solution was worse than that of each of the 10 restarts of simulated annealing.

There are a variety of ways in which the method in this paper can be extended. First, the analyses in this paper were restricted to the case of a single core; however, as noted in [6], there is the potential for multiple cores. Although the consideration of multiple cores increases the computational demand because of the presence of more clusters, the general computational scheme for correlation between two binary vectors is unaffected. Second, we reiterate that the computational scheme presently incorporated in the heuristic is limited to binary networks. As noted previously, the proposed heuristic was explicitly designed to capitalize on rapid computation of the correlation between binary vectors. For real-valued networks, sum-of-squares information would need to be stored for dynamic updating of the correlation measure; however, the basic structure of the simulated annealing heuristic could remain the same.
